# Strategies for Restructuring Dietetics Education Programs to Improve Nutrition Equity in Indigenous Populations: A Narrative Review

**DOI:** 10.3390/nu16234136

**Published:** 2024-11-29

**Authors:** Pamela N. Klassen, Catherine B. Chan

**Affiliations:** 1 Department of Agricultural, Food and Nutritional Sciences, University of Alberta, Edmonton, AB T6G 2R3, Canada; pamela2@ualberta.ca; 2 Department of Physiology, University of Alberta, Edmonton, AB T6G 2H7, Canada

**Keywords:** dietetics, curriculum, colonialism, Indigenous, First Nations, Aboriginal and Torres Islanders, Maori, American Indians and Alaska Natives, indigenization, decolonialization

## Abstract

Background/Objectives: Particularly in racially and ethnically diverse countries, the necessity of providing individualized care to people seeking diet advice is increasingly recognized and embedded in practice guidelines. Some jurisdictions have a history of colonization with subjugation and marginalization of the Indigenous population, which has led to serious health inequities. One overarching strategy to reduce health inequities is to provide education through a decolonizing lens, so that graduate healthcare professionals, such as dietitians, have a better understanding of how to mitigate colonial attitudes, racism, stereotyping and other behaviours, thereby improving health equity. This review aims to summarize and evaluate educational strategies to decolonize dietetics training programs. Methods: A narrative review was conducted. Results: Professional dietetics organizations in Canada, Australia and New Zealand have incorporated Indigenous-specific outcomes into their standards of practice. Six primary research studies were reviewed, two each from Australia, New Zealand and the United States. The strategies developed include reviewing curriculum content, providing experiential learning opportunities and identifying barriers to the participation of Indigenous students in dietetics programs. Lack of engagement of Indigenous persons in curriculum development, planning and evaluation of efforts is a gap that needs to be addressed. Conclusions: Meeting practice standards and closing the health equity gap for Indigenous peoples require additional research and implementation into practice.

## 1. Introduction and Rationale

The United States of America (USA), Canada, Australia and New Zealand are four major high-income countries with a colonial history, beginning in the 1500s with mainly British (or other European) populations settling lands already inhabited by Indigenous peoples with their own rich cultures. While the details may differ, in all four countries, the Indigenous peoples were (and still are) subjected to displacement from their traditional lands, loss of autonomy, overt and covert racism and attempts to assimilate them into the dominant society. Indigenous peoples’ experiences of historical and ongoing colonialism have created great harm, including a lower standard of living, high prevalence of chronic diseases [1] and enduring health inequities, leading to a shorter life expectancy in Canada [2], the USA [3], Australia [4] and New Zealand [5] (Table 1).

An important strategy to reduce health inequities is to provide education to students in healthcare professional programs that addresses the underlying causes. This should be performed using decolonizing practices, so that graduates of these programs, such as dietitians, have the knowledge, attitudes, skills and confidence to mitigate colonial attitudes, racism, stereotyping and other behaviours. This review aims to summarize and evaluate educational strategies to decolonize dietetics training programs with the ultimate goal of improving health equity.

In Canada, an overarching mandate to redress disparities related to colonialism is the Calls to Action, published in 2015 following a class action lawsuit (the Indian Residential Schools Settlement Agreement) brought by Indigenous peoples against the Government of Canada. The result of the lawsuit was the establishment in 2007 of the Truth and Reconciliation Commission (TRC) [6]. Indigenous perspectives on the history of the Residential School system were brought out into the open and 94 steps to address the societal inequities and racism faced under the colonial system were outlined in the Calls to Action, of which at least 5 demand change in the medical and education systems:

Action #18 calls for all levels of government to “acknowledge that the current state of Aboriginal health in Canada is a direct result of previous Canadian government policies…”.

Action #19 calls for the federal government to “…close the gaps in health outcomes between Aboriginal and non-Aboriginal communities”.

Action #22 calls on those who can effect change within the Canadian healthcare system to recognize the value of Aboriginal healing practices and use them in the treatment of Aboriginal patients in collaboration with Aboriginal healers and Elders where requested by Aboriginal patients.

Action #23.i. is to “increase the number of Aboriginal professionals working in the healthcare field”.

Action #23.iii. is to “provide cultural competency training for all healthcare professionals”.

Action #24 calls upon “medical and nursing schools to require all students to take a course dealing with Aboriginal health issues, including the history and legacy of residential schools, the United Nations Declaration on the Rights of Indigenous Peoples, Treaties and Aboriginal rights, and Indigenous teachings and practices. This will require skills-based training in intercultural competency, conflict resolution, human rights, and anti-racism”.

Readers interested in reconciliation and related activities ongoing in the countries covered by this review are referred to the National Centre for Truth and Reconciliation (Canada) [24], Reconciliation Australia [25], the Treaty of Waitangi [26] and the Waitangi Tribunal (New Zealand) [27]. The USA’s Indian Self-Determination and Education Assistance Act passed in 1975 is arguably that country’s guiding document on increasing autonomy for Indigenous peoples, including for social and health services [28]. Some states may have bodies to address Truth and Reconciliation, e.g., Washington State [29].

One lasting consequence of colonization is present-day health disparities that include nutritional inequities. It must be acknowledged that the poor health outcome data in Table 1 for Indigenous peoples do not arise from their ethnicity but rather from the fallout of colonialism (Call to Action #18). Across healthcare settings, the provision of food, nutrition education and medical nutrition therapy are the responsibility of professional dietitians, who are regulated health professionals trained through nationally accredited university programs. They often work in multi-disciplinary teams and directly interface with Indigenous peoples and communities to prevent or manage nutrition-related chronic diseases and optimize nutritional intake, thus having the opportunity to improve health equity through their actions. Another consequence of colonialism is low participation of Indigenous peoples in healthcare professions. Although data on dietitians who identify as Indigenous are scarce, research found that in the USA, 0.3% of members of the Academy of Nutrition and Dietetics were American Indian/Alaska Natives [30].

Considering the Canadian Calls to Action and the prevalence of nutrition-related health inequities in Indigenous populations across nations, this review will first set the context of post-colonial society, using Canada as an exemplar, to describe colonial actions that contributed to the current environment regarding nutrition-related health inequities. Next, we will summarize standards of practice in dietetics related to health equity and Indigenous health as set out by regulatory bodies in each country of interest. Finally, we will examine published research undertaken in the USA, Canada, Australia and New Zealand to understand interventions applied to develop a culturally competent dietetics workforce with strong representation by Indigenous peoples, focusing on the following question: What actions have been implemented/evaluated to ‘Indigenize’ dietetics training?

## 2. Methods

Given the complex topic of improving Indigenous health equity through the dietetics profession, we elected to compose a narrative review as a meaningful overview of the background, current state and potential actions to move forward. The peer-reviewed literature was searched alongside the grey literature to describe the historical context of colonial practices that impacted food and nutrition equity for Indigenous peoples. The grey literature was searched for regulatory standards related to health equity or Indigenous health in dietetics programs to ascertain the current situation. The peer-reviewed literature was searched for primary studies representing actions that have been taken within dietetics education to address Indigenous health inequities. These studies (1) were undertaken in Canada, New Zealand, Australia or the USA; (2) were specific to undergraduate or graduate dietetics training or included dietetics students; (3) described an action taken with the intent to decolonize, indigenize, improve students’ Indigenous knowledge, raise competence or similar; and (4) reported an evaluation or results of the action. Hand searching was undertaken to ensure that significant concepts were not missed.

## 3. Colonial Practices Impacting Food and Nutrition: The Example of Canada

Among the policies and practices that the Canadian federal government and other institutions used to exert control over Indigenous nations, several directly involved food-related actions. Banning traditional cultural practices and ceremonies, many of which involved food [31], or displacement from traditional lands, thereby removing them from their traditional food supply [32], both contributed to nutritional inequity for Indigenous peoples. Creation of the Residential School system forcibly removed children from their families. At many residential schools, food for the children was low in both quality and quantity, causing constant hunger, malnutrition and susceptibility to disease [33]. Boys were taught farming while the girls learned to cook European-style meals [32], rather than traditional hunting/gathering and cooking methods. Moreover, under government auspices, research was carried out by nutrition scientists in Indigenous communities and residential schools on malnourished individuals without their consent [33]. Although some of the root causes of malnutrition were correctly identified by these early researchers, the outcomes were often attributed to the First Nations people and not to the actions of the Canadian government or churches running the residential schools [33]. The horrific discoveries of unmarked graves of hundreds of First Nations children across Canada is evidence of increased susceptibility to disease, a hallmark of malnutrition.

In the present day, health inequities related to food, nutrition and displacement from traditional lands persist. Across the four countries, food insecurity is documented as being 50–100% higher in Indigenous populations than in the general or non-Indigenous population. Moreover, the prevalence of poor health outcomes from infant mortality to diabetes is generally higher in Indigenous populations (Table 1).

Barriers to equitable nutrition and nutrition care are complex and multi-faceted. Barriers to health equity include proximal, intermediate and distal determinants according to the Structural and Social Determinants of Indigenous People’s Health Framework [34]. Specifically with respect to nutrition, proximal and intermediate determinants include the remote locations of many Indigenous communities, which make transportation of food expensive, an issue exacerbated by poverty. Low education attainment may also hinder proper nutrition, as does lack of access to traditional food collection methods. Access to healthcare professionals is often limited in rural and remote communities, as is the representation of Indigenous people in healthcare professions [35]. However, distal factors related to colonialism also create important barriers to accessing healthcare in general: Eurocentric worldviews in both education and healthcare settings, lack of culturally safe and appropriate care, racism and social exclusion [34].

## 4. Results: Good Practices, Gaps, and Barriers in Dietetics Education Supporting Nutrition Equity for Indigenous Populations

As articulated by the United States Commission on Dietetic Registration, Academy of Nutrition and Dietetics [36], “Health equity is at the core of nutrition and dietetics practice where: (1) all individuals have the same opportunity and access to healthy food and nutrition; (2) Registered Dietitian Nutritionists (RDNs) advocate for a world where all people thrive through the transformative power of food and nutrition; and (3) RDNs work to accelerate improvements in health and well-being through food and nutrition”.

### 4.1. Standards of Practice That Support Indigenization and/or Decolonization of Curricula to Improve Health Equity

While definitions of “indigenization” and “decolonization” vary, Stein and colleagues [37] propose that indigenizing health curricula and training involves partnership with Indigenous people to support increased recruitment of Indigenous students and faculty and develop meaningful ways of integrating Indigenous ways of knowing. Some of these actions may be decolonizing, defined as addressing “the impact of historical and ongoing colonial dynamics in healthcare, identify and interrupt the root causes of health disparities and Indigenous Peoples’ negative experiences, support the Indigenous-led resurgence of Indigenous health practices, and foster more self-reflexivity among settler health practitioners about their complicity in colonialism” [37].

Standards for dietetics practice in Canada, Australia, New Zealand and the USA emphasize the importance of health equity, cultural safety and cultural competence (defined in Table 2). Three of the four countries’ dietetics standards of practice define health equity, with the USA and Canada using harmonized wording (Table 2). To achieve health equity, concepts such as cultural safety and competence of professionals needs to be addressed. New Zealand provides the most complete definitions for cultural safety and cultural competence. Interestingly, Canada’s definition of cultural safety speaks directly of freedom from racism, whereas New Zealand focuses on avoiding unconscious bias or stereotypes (Table 2). The USA and Australia do not provide definitions of cultural safety. These guiding documents should not serve solely as checklists but as an impetus for meaningful change that will necessarily be disruptive to the status quo [37].

While the USA does not have standards of practice that specifically refer to working with Indigenous peoples, Canada, Australia and New Zealand do (Table 3). Systemic racism toward Indigenous peoples is addressed by Canada (standard 2.03c) [41] and Australia (preamble, standard 1.5.4) [40]. Dietitians are expected to demonstrate awareness or recognition of Indigenous food environments, traditional foods and health practices in the context of dietetics practice in Canada, Australia and New Zealand [39,40,41]. Another commonality is that practice behaviours should include practicing in a culturally safe manner and advocating for changes that promote health equity (Table 3) [39,40,41].

### 4.2. Actions to Indigenize Dietetics Training

We identified six primary publications that represent efforts made to indigenize dietetics training, encompassing three broad actions. These actions were (1) reviewing and revising curricular content (Australia) [42,43], (2) implementing experiential learning opportunities (Australia, New Zealand) [44,45,46] and (3) identifying systemic barriers to Indigenous student participation in the profession (USA) [47,48]. Within the framework of these three actions, we will describe reported outcomes, summarize potential learnings, and identify potential gaps for future work.

#### 4.2.1. Curricular Content Review and Revision

Wilson et al. [42] used an action research process to consult members of a nutrition faculty alongside members of their university’s Indigenous Health faculty to review the existing dietetics curriculum, resulting in changes that, it was hoped, better prepare students to practice dietetics in Indigenous communities. An initial outcome of the process was an acknowledgement by existing nutrition and dietetics program staff that they lacked experience in this area and did not feel confident in their ability to teach students without perpetuating negative stereotypes of Indigenous people, and they cautioned against ‘tokenism’ in program changes. Second, faculty members were concerned that the addition of Indigenous content in each individual course of the program would not be sufficient to provide a foundation for students or a framework by which students could understand the larger context. The review process ultimately resulted in a recommendation for the addition of a semester-long Indigenous Health course requirement for dietetics students, which had been designed and used within other Health Sciences programs at the university. To complement this foundation, additional teaching across the dietetics program would be included in existing courses, specifically regarding issues of food, food choice and nutritional status in Indigenous communities. Gaps in this work include the lack of consultation with Indigenous advisors outside of academia and an absence of discussion on how existing staff would be supported to integrate the new material with confidence. Further, there was no articulated evaluation plan by which to measure the impact of the changes. Whether these gaps were addressed after publication or in recent years is unknown.

A second example of curricular content review is provided by McCartan et al. [43], in which a working group of the Department of Nutrition, Dietetics and Food at Monash University (Australia) set out to integrate the Health Curriculum Framework (the Framework) published by the Government of Australia into multiple courses in the first-year curriculum. The Framework included five cultural capability learning domains: respect, communication, safety and quality, reflection and advocacy, alongside seventeen learning outcomes for students [49]. The working group was supported by the Faculty’s Indigenous Health Curriculum Committee, which also provides mentorship and support for non-Indigenous educators in teaching Indigenous content. The resulting integrated curriculum included 14 Aboriginal health teaching activities and six assessment tasks within the first year of the program. McCartan et al. [43] used the Cultural Capability Measurement Tool to survey a cohort of students early in the first semester of their first year (prior to experiencing the new content) and compared their scores to week 9 of semester 2. Cultural capability scores significantly increased overall after the content exposure, with knowledge, attitude, commitment and confidence-related gains across the Framework domains of respect, communication, safety and quality, reflection and advocacy. This example supports a shift from simply adding cultural knowledge to curricula, to adding content and activities that enhance students’ cultural capability.

#### 4.2.2. Experiential Learning Opportunities

Three publications described the outcomes of experiential components in dietetics or allied health programs, generally implemented to increase graduate competency in Indigenous health and willingness to work in Indigenous health settings. Rae et al. [45] gathered informal feedback from a sample of six dietetics students who had visited an Aboriginal ArtsHealth Centre (once or multiple times) during their program. Students reported feeling uncertain about whether/when to approach nutrition topics with participants, highlighting the need for repeated visits to build rapport with Indigenous participants. They also identified a need for deeper understanding of Indigenous health disparities and historical context to be taught in their undergraduate curriculum. Svarc et al. [44] compared self-rated outcomes of dietetics graduates who had experienced an Aboriginal (Indigenous) health placement during their training with those who had not (*n* = 33 vs. *n* = 87). Graduates with placement experience felt more strongly that dietitians should learn about Indigenous history and culture, and that they had a personal responsibility to seek learning opportunities in Indigenous culture. They agreed more strongly that Indigenous people may define health differently than non-Indigenous people. Furthermore, those with placement experience were more confident in their ability to be culturally safe and were less apprehensive about interactions with Indigenous people. Semi-structured interviews with a subset of placement participants (*n* = 15) revealed perceived benefits to the placement including increased cultural empathy and gaining role models in Indigenous health practice. Interviewees reiterated the importance of gaining lived experience rather than theoretical training in Indigenous health.

Finally, Pullon et al. [46] reported student outcomes of the TIPE program in New Zealand, which placed allied health students in 5-week-long interprofessional education placements in rural areas [46]. While this was primarily an inter-professional educational model, subsets of the objectives were related to Indigenous health competency and rural/remote workforce development. The authors compared self-perceived learning between participating and non-participating students, finding higher self-perceived ratings for achievement of Indigenous health objectives, greater knowledge and incorporation of Indigenous culture and customs into practice and greater post-program participation in the rural/remote workforce. Related publications demonstrate positive feedback from placement providers [47].

Results from these three studies suggest that placement experiences contribute positively to developing a culturally competent Indigenous health workforce. Rae et al. highlight a need for systemic curricular changes followed by sustained, well-planned experiential learning, as opposed to short observations or exposure to Indigenous health programs [45]. Svarc et al. [44] and Pullon et al. [46] demonstrate that Indigenous health placements of longer durations (immersion in Indigenous health practice) are associated with improved cultural competence, comfort in Indigenous health practice settings, formation of practice mentor/role model relationships and improved willingness to work in Indigenous health settings after graduation. However, as the details of recruitment to the placement programs themselves were not described, we cannot be certain that those who chose to participate in placements did not at the baseline have higher cultural competence or sensitivity compared to those who did not participate. Further, primary outcomes were reported as perceived or rated by students themselves, and/or compared to students who did not participate in placements. No Indigenous perspectives were gathered; therefore, it is unknown whether the students’ perceived learnings translated to changes in their actual cross-cultural interactions or practice effectiveness. Cultural competency and associated measurable outcomes are not clearly defined, and thus program evaluations such as these rely heavily on perceptions.

#### 4.2.3. Identifying Systemic Barriers to Indigenous Student Participation

Two qualitative works identifying systemic barriers to Indigenous participation in dietetics or health programs were reviewed [48,50]. Cech et al. [48] began their inquiry with the theory that epistemological dominance in science, engineering and health fields would exclude Indigenous students, and sought to interview new Indigenous students in these fields to explore their experiences. Interviews with 44 students revealed first that the majority recognized tension between Indigenous and scientific epistemologies, and several noted hostility in their field toward Indigenous ways of knowing, also referred to as epistemological dominance. While, some students were able to marry the two ways of knowing in a complementary way, most recognized that their faculty or peers would not be able to do the same. Second, students experienced ‘delegitimation’ of Indigenous knowledge, including the absence of traditional Indigenous knowledge in textbooks or various teachings that Western scientists made discoveries that had long been known by Indigenous peoples. Further delegitimation was experienced when exploitation of Indigenous communities or resources was justified by the rigor of the scientific method. Finally, problematic pedagogical (teaching/learning) practices were identified by some students, including human or animal dissection, which in many cases is misaligned with Indigenous value systems. Students described having to explain this dilemma to their instructors, fearing or receiving poorer grades for not participating or experiencing stress associated with finding an alternative way of participating in the learning.

Hassel et al. [50] interviewed ten Indigenous scholars or elders and five non-Indigenous nutrition scholars to understand their perceptions of barriers to Indigenous participation in food, nutrition and health programs. By allowing the interviewees to guide the direction of the conversations, they garnered rich results, which highlight issues that likely apply across many disciplines in the health sciences. First, academic refusal to either acknowledge colonization and land dispossession or to take responsibility for the repercussions was identified as a primary barrier, rooted perhaps in this being felt as a threat to ‘the system’ or the success of the current academic faculty. Cultural disrespect or the perception that Indigenous people may have lower intelligence or gifting was also identified as a cause of significant error. Second, respondents discussed the problem of ignorance or invisibility of Indigenous knowledge in the discipline, and the refusal of Western science to look to Indigenous knowledge as another way of thinking that is necessary for a complete understanding of the natural world—referred to as disciplinary ‘blind spots’. Finally, responses highlighted the systemic disconnect between Western science/scientists and spirituality. As spirituality is considered by many Indigenous people to be inextricably bound to science, respondents felt it was an essential aspect of food and health for Indigenous people but was discarded or ignored in scientific disciplines.

## 5. Discussion

Canada’s Truth and Reconciliation Commission [6] called for change in the medical and education systems to redress Indigenous health disparities related to colonialism. While these calls to action were developed in the Canadian context, they can be applied across nations in which efforts are being made to address similar disparities. Each call has specific implications for the training of nutrition and dietetics professionals, and in several cases, they are congruent with Indigenous-specific standards of practice for dietetics in Canada, Australia and New Zealand. Accredited educational programs for dietitians are now responsible for mandating opportunities and curricular elements that not only enhance contextual understanding among dietetics students but also develop competence in culturally safe practice, application of traditional food and healing practices, engagement and collaboration with Indigenous communities and advocacy to advance health equity.

Our review identified literature from Australia and New Zealand in which efforts were primarily focused on enhancing dietetics students’ cultural understanding or cultural capability through curricular review or experiential learning opportunities. A well-planned program shift from knowledge acquisition to capability development described by McCartan et al. [43] included planned Aboriginal health teaching activities and assessment tasks in the first year of a dietetics program. This revision resulted in significant increases in self-rated cultural capability across domains. Similarly, evaluations of Indigenous health placements demonstrated improved cultural safety, ability to incorporate Indigenous culture or customs into practice and overall confidence in working with Indigenous peoples [44,45,46]. A significant gap in these works was the absence of evaluation based on Indigenous patient experiences, which may have presented different results than learner self-evaluation.

Beyond curricular revision and experiential learning, which are primarily focused on developing a culturally competent non-Indigenous health workforce, dietetics education programs are also called to increase the number of Indigenous healthcare professionals. This action requires identifying and addressing barriers to Indigenous participation in healthcare training programs. Interestingly, the literature identified in this area [48,50] was from USA, and it does not specifically define dietetics standards of practice for Indigenous healthcare. Learnings were focused on the importance of legitimizing Indigenous knowledge and ways of knowing, respecting Indigenous resources in research and adapting pedagogical practices to resolve misalignment between Western scientific methods and Indigenous values or customs. The strengths of these results lie in the strong representation of Indigenous voices and congruence of results between the two publications.

Our results should be considered alongside the ideas and perspectives presented in other recent works. In the Canadian context, Riediger et al. (no date) undertook gatherings/consultations in 2021 to help inform a Canadian dietetics program re-design, publishing the results in a white paper [51]. The importance of integrating specific Indigenous content, self-reflective exercises, Indigenous voices and experiential opportunities (to collaborate with communities) emerged clearly. Stein et al. (2023) emphasize the importance of confronting how dietetics has been and is complicit in actions that lead to food and nutrition inequities for Indigenous people, and caution that curricular changes must not be superficial or tokenistic. They call on settler scholars and healthcare professionals to work toward deep transformation in dietetics training programs, which will require stamina and relational commitment to the process [37]. To support the shift from ideas to action, Stein et al. also present a series of self-assessment questions for dietetics programs to consider when assessing the current state, barriers to change and involvement of Indigenous peoples in the program. It should be noted that many of the issues related to training in dietetics are similar to those faced by other health professions, for which there is a much larger literature base that could help support curricular transformations in dietetics. Some Canadian innovations in medical and nursing training programs have recently been reviewed [52].

In addition to the gaps in research highlighted, this review identified additional limitations. Although engagement and partnership with Indigenous people and communities is considered the best practice when developing Indigenous curriculum content and experiential learning, not all of the reviewed papers adopted this ideal. Furthermore, although planning and implementation is described, evaluation is limited and, as noted, does not include Indigenous perspectives. In the current work, we elected to focus on published work, but are cognizant that there is likely a wealth of unpublished information that might be uncovered by a thorough review of program websites and other materials. We recognize that this work, as a narrative review, may not contain all the relevant literature on this topic.

## 6. Conclusions

In order to break down the walls created by enduring colonial hierarchies in the realm of healthcare, implementing strategies in curricula and educational programming for dietetics students that will improve health equity requires the institutions offering dietetics programs to act both collectively and individually. Certainly, many activities have occurred at policy and instructional levels that have not been formally published. In this review, in the context of dietetics training in Canada, Australia, New Zealand and the USA, we summarized several actions that have been taken to bring meaningful change within dietetics programs. Creation of Indigenous-specific standards of practice, curricular review and revision, careful planning of experiential learning opportunities and addressing barriers to Indigenous participation in dietetics programs are all actions that must be accomplished through meaningful engagement and collaboration with Indigenous people and communities. Much work is required to ensure that program revisions are not tokenistic and ultimately balance the work of equipping a non-Indigenous health workforce while also developing a strong Indigenous health workforce. Finally, actions taken in this area and the associated learnings must continue to be shared in the literature to support the ongoing work of restoring health equity to Indigenous populations through decolonization and indigenization of nutrition and dietetics training.

## Figures and Tables

**Table 1:** Population representation and health inequities among Indigenous peoples.

		Canada	Australia	New Zealand	USA
Official designation		First Nations (FN), Inuit and Metis	Aboriginal and Torres Islanders (jointly termed First Nations)	Maori	American Indian and Alaska Natives (AIAN)
Population (%)					
		5.0 [6]	3.8 [7]	17.8 [8]	2.1 [9]
				
Life expectancy (y)	Comparator *	M 81.4, F 87.1	M 80.6, F 83.8	M 80.9, F 84.4	M & F 78.9
	Indigenous	FN M 72.5, F 77.7 Metis M 76.9, F 82.3 Inuit M 70.0, F 76.1 [2]	M 71.9, F 75.6 [4]	M 73.4, F 77.1 [5]	73.1 [10]
Infant mortality (per 1000 live births)	Comparator	3.7	3.5–2.9	3.8	4.5
	Indigenous	FN 8.5 Metis 7.0 Inuit 14.4 [11]	6.8–4.3 [12]	5.7 [13]	9.1 [14]
Diabetes prevalence (%)	Comparator	6.8	4–13	4.7	11.0
	Indigenous	FN 19.0 Metis 9.9 Inuit 4.7 [15]	22–32 [16]	7.5 [17]	16.0 [18]
Household food insecurity (%)	Comparator Indigenous	22.9 36.8 [19]	4–13 22–32 [20]	15.6 28.6 ** [21]	13.5 [22] 46 [23]

Abbreviations: M, male; F, female; FN, First Nations; AIAN, American Indian and Alaska Natives. * Comparator is either general population, non-Indigenous population, European/other or White (non-Hispanic) population. ** New Zealand data are for the proportion of children living in food-insecure households.

**Table 2:** Glossary of terms from the standards for dietetics practice from each country [38,39,40,41].

Term	Country	Definition
Regulated professional dietitian	Canada Australia * New Zealand USA	Registered Dietitian (RD) Dietitian Registered Dietitian Registered Dietitian Nutritionist (RDN)
Health equity	Canada and USA	Equity is the absence of avoidable, unfair, or remediable differences among groups of people, whether those groups are defined socially, economically, demographically or geographically or by other means of stratification. Health equity implies that ideally everyone should have a fair opportunity to attain their full health potential and that no one should be disadvantaged from achieving this potential.
	New Zealand	Health equity promotes fair and just health outcomes for all members of society. Relevant human rights include non-discrimination, equality, a level of education to fully participate in society, and a standard of living adequate for health. Efforts are made to remove structural barriers that create and maintain the gap between advantaged and disadvantaged groups.
Cultural safety	Canada	Cultural safety is an outcome based on respectful engagement that recognizes and strives to address power imbalances inherent in the healthcare system. It results in an environment free of racism and discrimination, where people feel safe when receiving health care.
	New Zealand	Cultural safety supports and promotes equity in health care. Given cultural diversity between and within cultural groups, only the client can determine the cultural appropriateness of their dietetic care. To achieve optimal outcomes, the dietitian needs to be culturally aware, sensitive and competent to identify and incorporate the client’s unique cultural beliefs, values and customs into their care. The dietitian does not rely on unconscious bias, assumptions or stereotypes that could adversely impact processes and outcomes. Any action that diminishes, demeans or disempowers the client’s cultural identity and well-being is considered to be unsafe cultural practice.
Cultural competence	New Zealand	Cultural competence is the dietitian’s ability to practise safely and effectively with a culturally diverse range of people. A dietitian needs to be aware of his/her own cultural biases and respect cultural differences. The dietitian accepts responsibility for acquiring and incorporating knowledge and skills to better understand members of other cultures and to develop therapeutic relationships with them for optimal client-centred care. Dietitians are committed to inclusivity, social justice and health equity.

* Australia does not provide definitions but does address cultural safety in the preamble (see Table 3).

**Table 3:** Standards specifically addressing Indigenous peoples in the context of dietetics practice.

Country	Standards
Canada [41]	Standard 1: 1.02c—Demonstrate awareness of Indigenous values and ways of knowing related to food environments; 1.05e Demonstrate awareness of the role of Indigenous traditional/country foods in dietary practices Standard 2: 2.03 Practice in a manner that promotes cultural safety; 2.03b Demonstrate awareness of Indigenous values and ways of knowing related to health and wellness; 2.03c. Demonstrate awareness of the ongoing impact of colonization/residential schools/intergenerational trauma/systemic racism on Indigenous peoples in Canada. Standard 4: 4.08 b. Demonstrate awareness of the availability and preparation of Indigenous traditional/country foods ^2^
Australia [40]	Preamble—Acknowledgement of the harms of colonialism on the Indigenous Peoples; Specific direction for dietitians to enhance their cultural safety and responsiveness for practice with Indigenous Peoples. Standard 1: 1.3.8 Recognises that whole systems—including health and education—are responsible for improving Aboriginal and Torres Strait Islander health, and collaborates with Aboriginal and Torres Strait Islander individuals and communities to advocate for social justice and health equity for Aboriginal and Torres Strait Islander peoples; 1.5 Demonstrates cultural safety and responsiveness; 1.5.4 Acknowledge colonisation and systemic racism, social, cultural, behavioural, and economic factors which impact Aboriginal and Torres Strait Islander peoples’ health outcomes and how this might influence dietetic practice and outcomes. Standard 4: 4.1.3 Engages in culturally appropriate, safe and sensitive communication that facilitates trust and the building of respectful relationships with Aboriginal and Torres Strait Islander peoples
New Zealand [39]	Preamble—Opens with a traditional greeting in the Maori language; Section 4—Cultural Responsiveness, which first affirms the Treaty of Waitangi as the nation’s founding document. It then indicates that the Dietitians Board is committed to upholding the principles embodied in the Treaty. Standard 2: 2.1.3. Respect Tikanga ^1^ when communicating with Maori; 2.2.2 Engage, motivate, empower and enable individuals, families/whanau and groups to achieve dietary behaviour and lifestyle change goals Standard 4: 4.3.4 Advocate for social justice and health equity for all groups including Maori; 4.3.5 Contribute to the reduction of social and health inequalities; 4.3.6 Apply Te Tiriti o Waitangi principles, Tikanga and Maori models of health (such as Te Whare Tapa Wha) to dietetic practice; 4.3.7 Be culturally responsive to client values, beliefs, and practices in relation to food, nutrition and health.
USA [38]	Standards related to health equity are written in a general sense and do not single out Indigenous peoples

^1^ A Maori word generally taken to mean ‘the Maori way of doing things’, referring to “the customary system of values and practices that have developed over time and are deeply embedded in the social context” [39]. ^2^ Traditional foods (also known as country foods) are foods that are locally available from natural resources and have cultural significance for Indigenous peoples in Canada. Traditional food is the preferred term for First Nations and Métis peoples, and country food is the preferred term for Inuit people [41].

## Dedication and Positionality of the Authorss

This work is dedicated to the late Honourable Murray Sinclair (1951–2024), the Commissioner of the Canadian Truth and Reconciliation Commission whose Calls to Action show us the way forward. Pamela Klassen is a Canadian settler dietitian without lived experience of the health inequities experienced by Indigenous people. She has practiced as an oncology dietitian and nutrition researcher on Treaty 6 Territory, Treaty 1 Territory, and in Tajikistan, CIS. Her contribution to this work was an exercise in learning and self-reflection, undertaken not from a position of expertise but from a posture of humility. Catherine Chan is a settler researcher and educator of western European ancestry who works and lives on Treaty 6 Territory. She grew up in British Columbia on the unceded territory of the Secwépemc peoples at Tk’mlúps (Kamloops). Catherine’s grandmother-in-law was Caroline McNabb, a Cree woman born into the George Gordon First Nation in Treaty 4 Territory (present-day Saskatchewan) in 1910 who, because of patriarchal policies of the Canadian Government, lost her Indian Status when she married a non-Indigenous man. Catherine is privileged to author this work in the hopes of furthering Truth and Reconciliation.
